# Survival functions for defining a clinical management Lost To Follow-Up (LTFU) cut-off in Antiretroviral Therapy (ART) program in Zomba, Malawi

**DOI:** 10.1186/s12911-016-0290-7

**Published:** 2016-05-05

**Authors:** Beth Rachlis, Donald C. Cole, Monique van Lettow, Michael Escobar

**Affiliations:** The Ontario HIV Treatment Network, Toronto, Canada; Division of Clinical Public Health, Dalla Lana School of Public Health, University of Toronto, Toronto, Canada; Division of Epidemiology, Dalla Lana School of Public Health, University of Toronto, Toronto, Canada; Dignitas International, Zomba, Malawi; Division of Biostatistics, Dalla Lana School of Public Health, University of Toronto, Toronto, Canada

**Keywords:** Antiretroviral therapy, HIV/AIDS, Retention, Survival functions, Malawi

## Abstract

**Background:**

While, lost to follow-up (LTFU) from antiretroviral therapy (ART) can be considered a catch-all category for patients who miss scheduled visits or medication pick-ups, operational definitions and methods for defining LTFU vary making comparisons across programs challenging. Using weekly cut-offs, we sought to determine the probability that an individual would return to clinic given that they had not yet returned in order to identify the LTFU cut-off that could be used to inform clinical management and tracing procedures.

**Methods:**

Individuals who initiated ART with Dignitas International supported sites (*n* = 22) in Zomba, Malawi between January 1 2007-June 30 2010 and were ≥ 1 week late for a follow-up visit were included. Lateness was categorized using weekly cut-offs from ≥1 to ≥26 weeks late. At each weekly cut-off, the proportion of patients who returned for a subsequent follow-up visit were identified. Cumulative Distribution Functions (CDFs) were plotted to determine the probability of returning as a function of lateness. Hazard functions were plotted to demonstrate the proportion of patients who returned each weekly interval relative to those who had yet to return.

**Results:**

In total, *n* = 4484 patients with *n* = 7316 follow-up visits were included. The number of included follow-up visits per patient ranged from 1–10 (median: 1). Both the CDF and hazard function demonstrated that after being ≥9 weeks late, the proportion of new patients who returned relative to those who had yet to return decreased substantially.

**Conclusions:**

We identified a LTFU definition useful for clinical management. The simple functions plotted here did not require advanced statistical expertise and were created using Microsoft Excel, making it a particularly practical method for HIV programs in resource-constrained settings.

**Electronic supplementary material:**

The online version of this article (doi:10.1186/s12911-016-0290-7) contains supplementary material, which is available to authorized users.

## Background

Operational definitions of Lost to Follow-up (LTFU) from antiretroviral therapy (ART) vary widely across settings, making comparisons across programs challenging [1]. The development and identification of standardized definitions of LTFU can inform cohort analyses [2, 3], program evaluations [4] and tracing mechanisms. While LTFU can be considered a general ‘catch-all’ category for patients who miss scheduled clinic visits or medication pick-ups [5], different definitions of LTFU within ART programs can have a significant impact on LTFU estimates over time [2, 4]. For example, in their 2013 study Shepherd et al., reported that cumulative estimates of LTFU varied widely, ranging from 22 % to 84 %; this variation was primarily dependent on the definition of LTFU that was applied [4]. This, in turn, has significant implications for program planning purposes such as the development of targeted strategies to improve retention.

Current methods to assess LTFU rates have relied largely on fixed time period cohort approaches, primarily through the use of retrospective cohort analyses. Attrition rates are often reported as proportions of patients who meet a specific outcome at various time points since ART initiation (e.g., 6 months, 12 months) [5–7], although definitions of outcome measures over a specified period of follow-up are generally unclear [8]. Importantly, there is no gold standard to measure retention in care, and different measures (e.g., missed visits, frequency of visits) have different advantages and limitations [9–11]. Generally, when such information is reported and available, patients who are known to have died, stopped ART or transferred out are generally excluded in reported LTFU rates. In addition to a general call for enhanced data on losses to follow-up [8], there has also been a push toward developing more evidence-based definitions [3, 12, 13] that minimize the misclassification of patients as LTFU. Related to this is the need for LTFU definitions that can be used for clinical management purposes. A clinical management definition of LTFU may be particularly useful in settings, such as Malawi, where limited resources are available for tracing. Indeed, high costs and a shortage of human resources necessary for finding missing patients have contributed to a backlog of patients needing to be traced in our setting. Identifying patients earlier who are risk of becoming lost and at risk of experiencing poor clinical outcomes has implications for patient retention overall. At the same time, if patients are prematurely classified as lost, limited resources may be used to trace individuals who ultimately will return on their own. Therefore, a LTFU definition specific to clinical management can inform clinicians of the optimum time to initiate the tracing process (in real time) in such a way that both patient and health system outcomes are maximized.

Chi et al. developed an empirical approach to determining a best-estimate definition of LTFU [3]. In their Zambian study, patients were classified as LTFU based on the number of days they were late for their most recent visit. They used weekly thresholds from ≥1 week to ≥26 weeks late. For each cut-off, they looked forward in their dataset to determine the proportion of patients who returned to care within the subsequent year. The cut-off that minimized the misclassification of patients as LTFU was considered the best-estimate definition, at least based on the available data. Receiver operating curves were plotted to demonstrate the cut-off that best determined whether a patient returned to care within the following year (i.e., the cut-off that minimized the misclassification of patients as LTFU). One of the limitations of this study however, was that only the patients’ last visit was included rather than all visits available [3] and thus not all available data was utilized.

Importantly, clinical decisions in resource-constrained settings are often made with incomplete and/or only partial information [14]. An analytic method, therefore, that is able to make use of all available data would be ideal in order to obtain the most complete picture possible. At the same time, LTFU definitions for clinical management purposes should be simple enough to calculate and interpret so that they can be applied by clinicians and program planners with limited statistical expertise and/or access to specific statistical software to make decisions about patient tracing in real time.

A time to event analysis offers one such method to determine the probability of a patient returning for a subsequent visit based on how late they already are for an expected visit. A time to event analysis studies the time it takes for an outcome to occur, i.e., time to death, time to disease [15, 16] or in this case time to a return. A time to event analysis is often called a survival analysis in the biostatistical literature. However, even when the outcome of interest is not death, one can still use this well-developed body of literature to study the time to non-death events. Cumulative Distribution Functions (CDFs), the complement of the survival function, are a fundamental way to define random variables. Essentially, they describe ‘Area Under the Curve’ functions [17, 18]. CDFs have been used and previously applied in multiple epidemiologic studies including those related to spatial analysis and environmental epidemiology [19–21], surveillance and complex models of disease transmission [22, 23], and studies involving clinical decision-making [14, 24–27]. Related to the CDF is the hazard function, which provides a measure of risk and plots the probability of an event occurring at or over a period of time given that the event has not already occurred [15, 28]. The hazard function in the present analysis provides insight regarding the proportion of patients who return each 1-week interval, of those who have yet to return. As such, clinical staff can use this information to determine when tracing should be initiated (e.g., immediately vs. waiting another week).

CDFs and hazard functions have numerous advantages; for example, they represent data visually in a relatively straightforward and intuitive manner [20]. They can make use of all available data, and as a result tend to present distributions as completely as the dataset allows [15, 29, 30]. Since the CDF is 1 minus the survival function, the variance of the CDF is the same as that of the survival function. This can be calculated through standard methods including Greenwood’s formula [31]. One can refer to most standard books on statistical survival analysis (e.g., Cox & Oakes, 1984) [32].

In the analysis reported here, the CDF and the hazard function were both plotted and examined to determine the probability that a patient would return each week, given that they had not yet returned, in order to identify the LTFU cut-off that could best inform clinical management and tracing procedures.

## Methods

Dignitas International (DI), a Canadian non-governmental organization, has worked in partnership with the Malawi Ministry of Health (MOH) since 2004 to support delivery of comprehensive HIV/AIDS care in the Zomba District, one of the most densely populated districts in Malawi (population: 670,500). District HIV prevalence is approximately 14.5 % although estimates within the district vary by location and population group [33]. DI supported the Malawi MOH to establish a tertiary referral HIV clinic at Zomba Central Hospital in 2004, and since 2006 has also supported the Zomba District Health Office to integrate HIV-related services into existing primary health services at decentralized health centres throughout the district [34]. As per Malawian MOH guidelines, each patient who starts ART is given a unique treatment unit ART registration number. This number is written on a paper-based patient card called a Master Card and put into the electronic ART register for staff’s use. All baseline registration data is entered at the time of ART initiation. At each follow-up (FU) visit, patient data is documented on the Master Card. Following initiation, patients are asked to return 2 weeks later and then monthly for the first 6 months. After 6 months, patients may be asked to return every 2–3 months depending primarily upon provider assessments and drug availability [34, 35]. Patients are therefore given a 2-week, 4-week, 8-week or 12-week supply of ART depending on their expected frequency of visits. Data collection on MOH standardized registers and Master Cards are completed by clinicians and health staff at baseline initiation and at each subsequent FU visit.

For each follow-up visit, a ‘days late’ value was determined. The expected return date was calculated by using the value for ART supply (in weeks) given at each FU visit. A patient’s actual return date was compared to their expected return date. A patient was considered late if they did not return at least 7 days after they were expected for a FU visit. This definition is consistent with the 7-days late value used to generate adherence proportions in the Malawi treatment guidelines [35]. As well, discussion with the clinical team in Zomba (personal communication Gabriel Mateyu and Dr. Kevin Bezanson, Dignitas International, Zomba Malawi November 29, 2012) supported use of this definition of *Late*.

Building on the work of Chi et al. described above [3], we sought to determine a clinical management LTFU cut-off. Individuals 15 years of age and over who initiated ART with Dignitas International (DI) supported sites including the Central hospital and various health centres in rural areas in Zomba, Malawi between 1 January 2007 and 1 July 2010 were eligible for inclusion. The analysis was limited to all follow-up visits where the patient was ≥1 week late. Similar to Chi et al., lateness was categorized using 1-week cut-offs from ≥1 week to ≥26 weeks late [3]. Therefore, this analysis was based on patients who were at least ≥1 week late and likely to have run out of ART, and thus at a high risk of becoming LTFU. Further, patients were excluded if they were known to have transferred out, stopped ART or died, based on the limited successful tracing completed [36]. Note we considered including known deaths as a competing event although we did not feel confident that we could accurately ascertain deaths given incomplete tracing data. Furthermore, only 70 deaths ascertained through tracing were available for this analyses and the median time from a last known visit date and death ascertainment date (through tracing) was 299.5 days. As in Chi et al., we limited our analyses to patients who started ART and had a FU visit scheduled at least 12 months prior to the end of the study period [3]. This helped to ensure that patients had adequate time to return to the clinic [37]. Furthermore, given that the risk of death is often highest in the first few months of treatment [38–41], allowed us to minimize the inclusion of patients who did not return to the clinic because they had died. As we could not ascertain deaths in our dataset, we may have overestimated LTFU estimates.

The DI main dataset, available and maintained in Microsoft ACCESS, is composed of 21 separate tables. Several queries were run in order to construct a single flat file dataset that contained all relevant data (baseline and FU visits) on patients meeting the inclusion criteria. Similar to previous studies utilizing this method [42] the data was presented over a wide range of cut-offs. At each 1-week cut-off, the proportion of patients who returned for a subsequent follow-up visit was identified along with 95 % confidence intervals. The confidence intervals were constructed using the variance of the survival function. Plots were generated in Microsoft Excel 2007 (See Additional files 1 and 2). The data used in this study was extracted from routine monitoring and evaluation data gathered as part of the DI/Malawi MOH ART program in Zomba, Malawi, using standardized national Master Cards and registers. As all analyses were performed with de-identified data that was extracted from routine programmatic information, patients did not provide individual written or verbal consent to participate in the study. Ethical approval for data use was obtained from the University of Toronto HIV Research Ethics Board and the National Health Sciences Research Committee in Malawi.

## Results

**Fig. 1:**
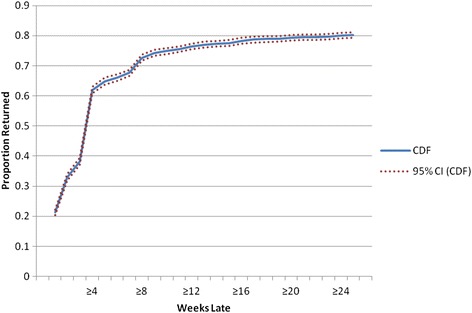
Cumulative Distribution Function to Estimate a Lost To Follow-Up Cut-off

In total, *n* = 4484 patients with *n* = 7316 follow-up visits were included. The number of follow-up visits included per patient ranged from 1 to 10 (Median: 1 visit). The median time on ART was 182 days (Interquartile Range: 70–336 days). Included patients were receiving care at *n* = 22 DI-supported sites. Approximately 40 % of patients were receiving ART at the Central Hospital (*n* = 1796) and 1929 (i.e., 43 %) were at least 9 weeks late for a visit. The CDF demonstrated that the majority of patients who were ≥1 week late and subsequently returned to care did so within the first 4 weeks of a missed visit, as per the initial sharp increase of the curve (see Fig. 1).

**Fig. 2:**
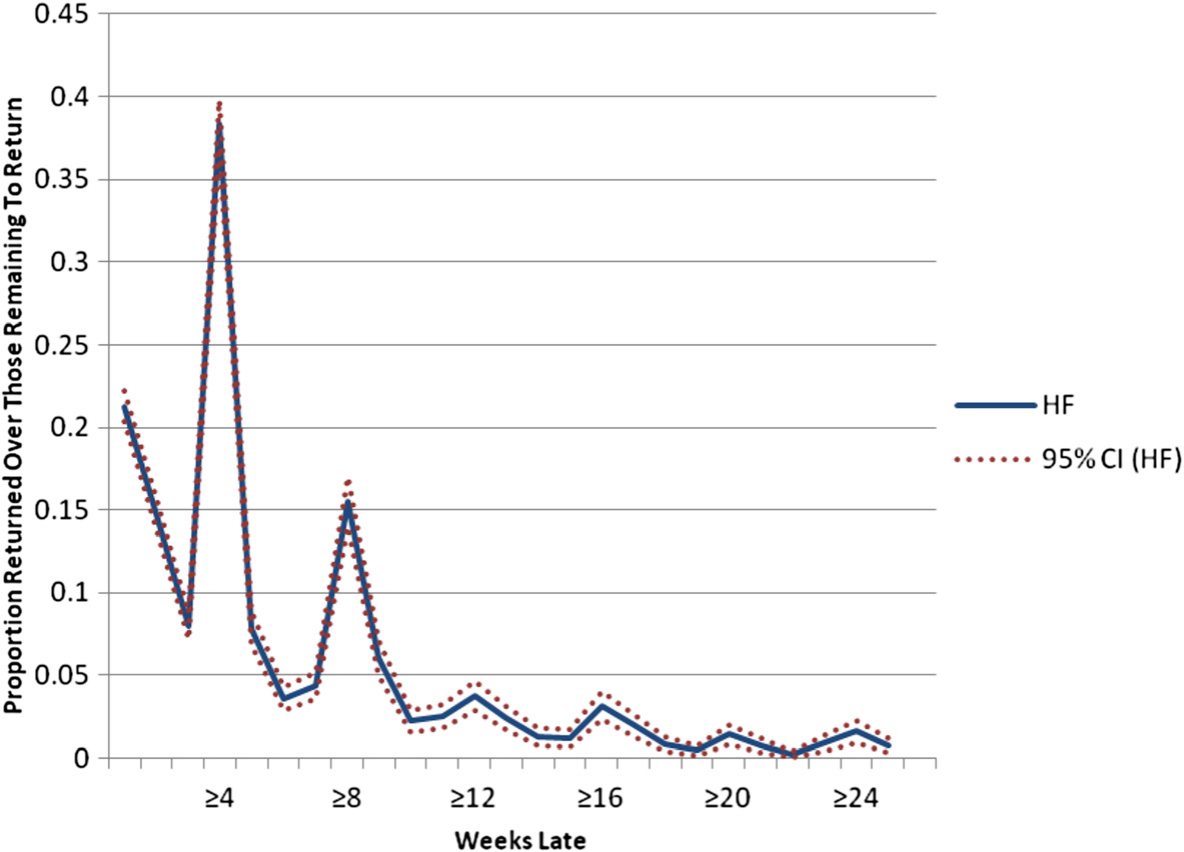
Hazard Function to Estimate a Lost to Follow-Up Cut-off

Although fewer, patients who were ≥1 week late continued to return even after being 4 weeks late as demonstrated by the more gradual increase in the curve until approximately the 9-weeks-late cut-off. The curve then begins to flatten out, suggesting that the proportion of patients who returned for a subsequent visit decreased once a patient was approximately 9 weeks late for a visit. The hazard function (Fig. 2) shows two main spikes in the proportion of patients who returned relative to those who had yet to return, roughly at 4-week intervals, at 4 weeks and again around the 8-weeks-late cut-off. As in the CDF, Fig. 2 demonstrates that after being at least 9 weeks late, the proportion of new patients who returned relative to those who had yet to return decreased substantially.

## Discussion

The clinical management LTFU definition of approximately 9 weeks identified in this study is relatively consistent with previous literature on losses to follow-up [3, 43, 44]. Chi et al. found that the use of an ≥8-weeks-late cut-off minimized the misclassification of patients as LTFU in their multi-site study in Zambia [3]. The LTFU cut-off in this study is also similar to what is currently used in Malawi to define patients who are LTFU (i.e., 2 months late for a scheduled visit) and this has implications for clinical management guidelines. At the time of the study (i.e., 2007–2010), the Malawian MOH guidelines indicated that home visits (following unsuccessful telephone call attempts) should occur *no later* than 14 days or 2 weeks after a missed visit [35]. In a parallel, exploratory analysis of Dignitas tracing data, among the 83 patients who were successfully traced (of the 232 with available data), the median number of days from the last visit to a successful trace date was 181.5 days, ranging from 159 days to 306.5 days, considerably longer than the guidelines at the time of this study [36]. Interestingly, the updated 2011 guidelines suggest that home visit attempts should be made *from* 2 weeks after a missed visit [45]. This subtle change in wording provides patients with more time to return and can thus reduce unnecessary tracing. Indeed, the functions presented indicate that most patients who are already 1 week late and do eventually return for a subsequent visit will return within the first 4 weeks of a missed visit. Currently, limited financial and human resources available for tracing at Dignitas ART sites has led to a backlog of patients waiting to be traced. The findings presented here suggest that in some cases, patients are being traced too prematurely following a missed visit.

While our study identified a LTFU definition for clinical management that made sense in our setting given the data and frequency of visits, it is important to note that the actual definition that we determined (i.e., 9 weeks) may not be appropriate or relevant in other programs and contexts [4]. Indeed, a universal definition or a gold standard measure applied across programs may not be appropriate given different program characteristics [4]. The present study demonstrates LTFU questions can be reframed as a time to event analysis. By reframing the question in this way and by using a well-known, simple, robust, nonparametric technique from this literature, we have found a strong and surprising result that has not, too our knowledge, been reported to date. In the future, we hope to continue modifying techniques from that literature in order to further understand LTFU issues. This would include using methods such as Cox proportional hazard models [46] or Poisson regression methods for life table analysis [47, 48]. Our goal was to introduce a simple methodology based on survival functions that can be used to estimate evidence-based cut-off for defining LTFU specific to clinical management. Investigators interested in examining factors related to lost to follow-up could consider the vast the well-developed literature and additional methods including survival models, Cox proportional hazards models and Poisson regression models. Furthermore, several standardized definitions may be useful depending on the population of interest [4, 11]. For example, a different definition for pre-ART patients may be warranted given different visit frequencies (e.g., no regular pharmacy visits). As a result, a longer LTFU definition may be more appropriate for pre-ART patients [12]. Definitions of LTFU relevant for different population groups should be explored further as they may have different cut-offs that are appropriate.

There are several limitations of using CDFs and hazard functions to establish clinical management LTFU cut-offs. The primary issue relates to the judgement involved in deciding which cut-off is meaningful for categorizing LTFU for clinical management purposes given available data. As noted above, different definitions of LTFU can have a significant impact on LTFU estimates [2, 4]. While the shape of the functions provides some insight (e.g., where the curve flattens in the CDF, spikes in the hazard function), the weeks-late value used to define LTFU in the present study is still a judgement made without a formal set of criteria. The spikes, for example, in the hazard function may be reflective of the expected frequency of visits and the ART supply given (e.g., 4 or 8 week supply). It is worth noting, however, that the visual appraisal of both functions suggests a ≥9-weeks-late clinical management LTFU cut-off. This method therefore has strength in that it can act as a tool for triaging [37] patients for active tracing.

As in other methods, data preparation is a necessary step to ensure that the curves can be generated (See Additional file 1). To establish whether a patient was late for a visit, the ART supply given (in weeks) at their most recent visit was used to determine when they would be expected for their next visit. This information may not always be available given the lack of tracking data and, therefore, the date of the next scheduled visit may also not be reported. Interestingly, 98 % of ART clinics in one East African multi-site study did report recording the next expected appointment date, although under a third actually compared the expected appointment date with the actual return date [1].

Missing data is often a challenge in clinical databases. Generally, patients are said to be censored when information on time to event (of interest) is not available for all participants, including those whom become LTFU [49]. As a result, LTFU is often considered a non-informative censoring event in cohort analyses [2, 50]. In this program of research, however, LTFU is the primary outcome of interest. The reasons for censoring the examined data therefore, are mostly not available, as incomplete follow-up occurs for many patients without adequate resources to enable full or complete follow-up. While death is a competing risk for becoming LTFU (i.e., patients who die are no longer at risk for becoming lost), given incomplete data on known deaths (primarily due to poor ascertainment through tracing) for individuals who had missed their visits, we did not explore death as a competing risk in the present analysis. This is an important limitation as this can lead to overestimations of LTFU [51] even those used for clinical management purposes. Death reporting is neither compulsory nor enforced legally in Malawi [52]. As a result, deaths are generally under reported; this makes linkages with health surveillance and clinical tracking data challenging. However, it is worth noting that while some of those lost to follow-up probably died (about one-third of those successfully traced in this program) effectively taking them out of the denominator of those ‘at risk’ of returning for an additional follow-up visit. However, even if 20 % of those LTFU (larger than the 17 % in Malawi overall) are removed from the denominator i.e. censored, they are unlikely to affect the fundamental shape of the curves substantially, as per the stratified analyses. Indeed, numerous studies have demonstrated higher rates of mortality in the first few months after ART initiation [38–41]. However, while we could not fully account for the impact of deaths in this study, we sought to minimize the number of patients who died in this dataset by excluding patients who had not initiated ART at least 12 months prior to the end of the study although it is important to note that each patient in this study had at least 12 months to return to FU visit as in Chi et al. [12]. As we did not have a specific time limit that patients had to return by in order to be defined as clinically LTFU, there are variable times to return. For example, a patient with a scheduled follow-up visit early in the study period of interest has a longer opportunity to return versus a patients who scheduled FU visit was approximately 12 months from study endpoint (i.e., June 30 2009). Furthermore, a patient may still return after the study period and therefore only have experienced a gap in care rather than be truly LTFU. As visit-level data (versus patient-level) was used to establish clinical management LTFU cut-offs, there may be a differential contribution to data from different patients, as they may have different numbers of follow-up visits included. While it is worth noting that the median number of follow-up visits was 1, the number of follow-up visits ranged from 1 to 10. The small number of follow-up visits per patient may stem from our inclusion criteria of being at least 7 days late for an expected follow-up visit. In a previous analysis [53], we noted that a patient returns to the clinic within 6 days of an expected follow-up visit and are only 7 days late in approximately 17 % of follow-up visits. This may partially explain why the median number of follow-up visits per patient was 1. Regardless, we did not account for multiple follow-up visits per patient in the present study as we were focusing on clinician’s decision about a particular follow-up visit.

CDFs provided a comprehensive presentation of data over a large range of cut-offs and offered guidance regarding the clinical management LTFU cut-off in this setting. The cut-off was further corroborated through the plotting of the associated hazard function. Importantly, there is no gold standard definition of retention and our clinical management LTFU definition may not be appropriate in other settings given that program characteristics and populations of interests can widely across programs and settings [4]. However, having a sense of the proportion of patients who return weekly can help clinical staff, regardless of the program or setting, to predict the number of patients to be expected each week. For example, at the 3 weeks late cut-off, it is expected that approximately 40 % of the remaining patients who are late and who will return will show up during the following week. Clinical staff can then decide the value in waiting another week to start tracing versus implementing tracing immediately.

## Conclusions

Flexible and informative, the simple functions plotted here did not require advanced statistical expertise and were created using Microsoft Excel, making it a particularly practical method for HIV programs in resource-constrained settings. As a result, they should be considered additional tools for ART program monitoring specialists and clinical managers in Malawi and other resource-constrained settings. In addition to identifying other clinical management definitions that may be relevant for different population groups (e.g., pre-ART patients), future studies should pilot the use of survival functions and explore the acceptability of this tool among program managers as well as determine their general utility.

### Consent for publication

Not applicable.

### Availability of data and materials

The dataset supporting the conclusions of this article is included within the article (and its additional file).

## Additional files

Additional file 1: Instructions for Using Survival Functions to Generate LTFU Cut-Offs. (DOC 26 kb) Additional file 2: Excel Spreadsheet Demonstrating Calculations of Hazard and Cumulative Distribution Functions. (DOC 69 kb)

## Abbreviations

ART: antiretroviral therapy
CDF: cumulative distribution function
DI: dignitas international
FU: follow-up (visit)
LTFU: lost to follow-up
MOH: ministry of health (Malawi)
WHO: World Health Organization
