# Supplementary material for: Survival functions for defining a clinical management Lost To Follow-Up (LTFU) cut-off in Antiretroviral Therapy (ART) program in Zomba, Malawi
Source: BMC Med Inform Decis Mak. 2016 May 5;16:52. doi: 10.1186/s12911-016-0290-7 (PMC4857410; doi:10.1186/s12911-016-0290-7)
Supplement: Additional file 1: — Instructions for Using Survival Functions to Generate LTFU Cut-Offs. (DOC 26 kb) [file 12911_2016_290_MOESM1_ESM.doc]

**Additional File 1:**

**A. Instructions for Using Survival Functions to Generate LTFU Cut-Offs**

**Data Set Up and Preparation**

1. For each follow-up visit, determine the next expected date a patient will return (if possible, use the ART supply given at the most recent visit);

2. Compare the expected return date with the actual return date. For patients who did not return, use the study end point.

3. Create a variable that reflects the difference between the expected and actual return (i.e., ‘days late’)

4. Organize the variable selected in item 3 above into weekly thresholds (i.e., ≥1 week late, ≥2 weeks late)

5. Choose a minimum days late that would be reflective of LTFU (i.e., at least 1 week late)

6. Determine the total number of visits meeting each threshold criteria for days late (e.g., number of visits where the patient was at least 1 week late, 2 weeks late, 3 weeks late etc. This is the number at the start of the period (rj)

7. Look forward in your dataset to determine the number visits where the patient returned for a subsequent visit in the subsequent week/interval (dj)

8. Calculate the Hazard Function (HF): proportion of visits where the patient returned for each weekly interval: dj/rj.

9. Calculate the standard error associated with the HF calculated in item 3 for each interval: √(HF*(1-HF)/rj)

10. Calculate the 95% Confidence intervals (95% CI): HF±1.96*se(HF)

11. Calculate the Survival Function (SF): For the first interval: 1- HF. For each subsequent interval: Survival Function for previous interval*(1-HF).

12. Calculate the Cumulative Distribution Function (CDF): 1-Survival Function

13. To calculate the standard errors associated with the CDF, first calculate the Variance (log Survival Function): for the first interval: dj/(rj*(rj-dj). The Var(logSF) then continues to incorporate the Var(logSF) of the previous interval: Var(logSF of previous interval) + dj/rj*(rj-dj)

14. Calculate the standard error associated with the CDF: Survival Function*√Var(logSF)

15. Calculate 95% CI for the CDF: CDF±1.96*se(CDF)
