# Supplementary material for: Survival functions for defining a clinical management Lost To Follow-Up (LTFU) cut-off in Antiretroviral Therapy (ART) program in Zomba, Malawi
Source: BMC Med Inform Decis Mak. 2016 May 5;16:52. doi: 10.1186/s12911-016-0290-7 (PMC4857410; doi:10.1186/s12911-016-0290-7)
Supplement: Additional file 2: — Excel Spreadsheet Demonstrating Calculations of Hazard and Cumulative Distribution Functions. (DOC 69 kb) [file 12911_2016_290_MOESM2_ESM.doc]

Excel Spreadsheet Demonstrating Calculations of Hazard and Cumulative Distribution Functions

| Weeks Late | Total n visits at beginning of next period | rj: Number at start of period | dj: number of returns in interval | HF | se(HF) | 95% LCI (HF) | 95% UCI(HF) | SurvF | CDF | Var(logSurv) | se(CDF) | 95% LCI(CDF) | 95% UCI(CDF) |
| --- | --- | --- | --- | --- | --- | --- | --- | --- | --- | --- | --- | --- | --- |
| ≥1 | 5759 | 7316 | 1557 | 0.212821 | 0.004785 | 0.203442 | 0.2222 | 0.787179 | 0.212821 | 3.69545E-05 | 0.004785 | 0.203442 | 0.2222 |
| ≥2 | 4911 | 5759 | 848 | 0.147248 | 0.004669 | 0.138096 | 0.1564 | 0.671268 | 0.328732 | 6.69378E-05 | 0.005492 | 0.317967 | 0.339496 |
| ≥3 | 4518 | 4911 | 393 | 0.080024 | 0.003872 | 0.072436 | 0.087613 | 0.617551 | 0.382449 | 8.46502E-05 | 0.005682 | 0.371313 | 0.393586 |
| ≥4 | 2786 | 4518 | 1732 | 0.383355 | 0.007233 | 0.369178 | 0.397533 | 0.380809 | 0.619191 | 0.000222251 | 0.005677 | 0.608064 | 0.630318 |
| ≥5 | 2567 | 2786 | 219 | 0.078607 | 0.005099 | 0.068614 | 0.088601 | 0.350875 | 0.649125 | 0.000252873 | 0.00558 | 0.638189 | 0.660061 |
| ≥6 | 2475 | 2567 | 92 | 0.03584 | 0.003669 | 0.028648 | 0.043031 | 0.3383 | 0.6617 | 0.000267354 | 0.005532 | 0.650859 | 0.672542 |
| ≥7 | 2367 | 2475 | 108 | 0.043636 | 0.004106 | 0.035588 | 0.051685 | 0.323537 | 0.676463 | 0.000285789 | 0.005469 | 0.665742 | 0.687183 |
| ≥8 | 2000 | 2367 | 367 | 0.155049 | 0.00744 | 0.140467 | 0.16963 | 0.273373 | 0.726627 | 0.000363313 | 0.005211 | 0.716414 | 0.73684 |
| ≥9 | 1879 | 2000 | 121 | 0.0605 | 0.005331 | 0.050051 | 0.070949 | 0.256834 | 0.743166 | 0.000395511 | 0.005108 | 0.733154 | 0.753177 |
| ≥10 | 1837 | 1879 | 42 | 0.022352 | 0.00341 | 0.015668 | 0.029036 | 0.251093 | 0.748907 | 0.000407679 | 0.00507 | 0.73897 | 0.758843 |
| ≥11 | 1791 | 1837 | 46 | 0.025041 | 0.003646 | 0.017896 | 0.032186 | 0.244806 | 0.755194 | 0.000421661 | 0.005027 | 0.745341 | 0.765047 |
| ≥12 | 1723 | 1791 | 68 | 0.037968 | 0.004516 | 0.029116 | 0.046819 | 0.235511 | 0.764489 | 0.000443696 | 0.004961 | 0.754766 | 0.774212 |
| ≥13 | 1681 | 1723 | 42 | 0.024376 | 0.003715 | 0.017094 | 0.031658 | 0.22977 | 0.77023 | 0.000458197 | 0.004918 | 0.76059 | 0.77987 |
| ≥14 | 1660 | 1681 | 21 | 0.012493 | 0.002709 | 0.007183 | 0.017802 | 0.2269 | 0.7731 | 0.000465723 | 0.004897 | 0.763503 | 0.782697 |
| ≥15 | 1640 | 1660 | 20 | 0.012048 | 0.002678 | 0.0068 | 0.017297 | 0.224166 | 0.775834 | 0.000473069 | 0.004876 | 0.766278 | 0.78539 |
| ≥16 | 1588 | 1640 | 52 | 0.031707 | 0.004327 | 0.023227 | 0.040188 | 0.217059 | 0.782941 | 0.000493036 | 0.00482 | 0.773495 | 0.792388 |
| ≥17 | 1557 | 1588 | 31 | 0.019521 | 0.003472 | 0.012717 | 0.026326 | 0.212821 | 0.787179 | 0.000505574 | 0.004785 | 0.7778 | 0.796558 |
| ≥18 | 1544 | 1557 | 13 | 0.008349 | 0.002306 | 0.00383 | 0.012869 | 0.211044 | 0.788956 | 0.000510982 | 0.004771 | 0.779605 | 0.798306 |
| ≥19 | 1537 | 1544 | 7 | 0.004534 | 0.00171 | 0.001183 | 0.007885 | 0.210087 | 0.789913 | 0.000513931 | 0.004763 | 0.780578 | 0.799247 |
| ≥20 | 1515 | 1537 | 22 | 0.014314 | 0.00303 | 0.008375 | 0.020252 | 0.20708 | 0.79292 | 0.000523379 | 0.004737 | 0.783634 | 0.802205 |
| ≥21 | 1503 | 1515 | 12 | 0.007921 | 0.002277 | 0.003457 | 0.012385 | 0.20544 | 0.79456 | 0.000528649 | 0.004724 | 0.785302 | 0.803818 |
| ≥22 | 1500 | 1503 | 3 | 0.001996 | 0.001151 | 0.00026 | 0.004252 | 0.20503 | 0.79497 | 0.00052998 | 0.00472 | 0.785719 | 0.804221 |
| ≥23 | 1486 | 1500 | 14 | 0.009333 | 0.002483 | 0.004467 | 0.0142 | 0.203116 | 0.796884 | 0.000536261 | 0.004704 | 0.787664 | 0.806103 |
| ≥24 | 1462 | 1486 | 24 | 0.016151 | 0.00327 | 0.009741 | 0.02256 | 0.199836 | 0.800164 | 0.000547308 | 0.004675 | 0.791001 | 0.809327 |
| ≥25 | 1451 | 1462 | 11 | 0.007524 | 0.00226 | 0.003094 | 0.011954 | 0.198332 | 0.801668 | 0.000552493 | 0.004662 | 0.79253 | 0.810805 |
